# Parallel distributed networks resolved at high resolution reveal close juxtaposition of distinct regions

**DOI:** 10.1152/jn.00808.2018

**Published:** 2019-02-20

**Authors:** Rodrigo M. Braga, Koene R. A. Van Dijk, Jonathan R. Polimeni, Mark C. Eldaief, Randy L. Buckner

**Affiliations:** ^1^Department of Psychology, Center for Brain Science, Harvard University, Cambridge, Massachusetts; ^2^The Computational, Cognitive & Clinical Neuroimaging Laboratory, Hammersmith Hospital Campus, Imperial College London, London, United Kingdom; ^3^Athinoula A. Martinos Center for Biomedical Imaging, Massachusetts General Hospital, Charlestown, Massachusetts; ^4^Department of Radiology, Harvard Medical School, Boston, Massachusetts; ^5^Division of Health Sciences and Technology, Massachusetts Institute of Technology, Cambridge, Massachusetts; ^6^Department of Psychiatry, Massachusetts General Hospital, Charlestown, Massachusetts

**Keywords:** association cortex, default network, hippocampus, subiculum, UK Biobank

## Abstract

Examination of large-scale distributed networks within the individual reveals details of cortical network organization that are absent in group-averaged studies. One recent discovery is that a distributed transmodal network, often referred to as the “default network,” comprises two closely interdigitated networks, only one of which is coupled to posterior parahippocampal cortex. Not all studies of individuals have identified the same networks, and questions remain about the degree to which the two networks are separate, particularly within regions hypothesized to be interconnected hubs. In this study we replicate the observation of network separation across analytical (seed-based connectivity and parcellation) and data projection (volume and surface) methods in two individuals each scanned 31 times. Additionally, three individuals were examined with high-resolution (7T; 1.35 mm) functional magnetic resonance imaging to gain further insight into the anatomical details. The two networks were identified with separate regions localized to adjacent portions of the cortical ribbon, sometimes inside the same sulcus. Midline regions previously implicated as hubs revealed near complete spatial separation of the two networks, displaying a complex spatial topography in the posterior cingulate and precuneus. The network coupled to parahippocampal cortex also revealed a separate region directly within the hippocampus, at or near the subiculum. These collective results support that the default network is composed of at least two spatially juxtaposed networks. Fine spatial details and juxtapositions of the two networks can be identified within individuals at high resolution, providing insight into the network organization of association cortex and placing further constraints on interpretation of group-averaged neuroimaging data.

**NEW & NOTEWORTHY** Recent evidence has emerged that canonical large-scale networks such as the “default network” fractionate into parallel distributed networks when defined within individuals. This research uses high-resolution imaging to show that the networks possess juxtapositions sometimes evident inside the same sulcus and within regions that have been previously hypothesized to be network hubs. Distinct circumscribed regions of one network were also resolved in the hippocampal formation, at or near the parahippocampal cortex and subiculum.

## INTRODUCTION

The association cortex comprises parallel distributed networks involving temporal, parietal, frontal, and anterior and posterior midline regions (Doucet et al. 2011; Margulies et al. 2016; Power et al. 2011; Yeo et al. 2011; see also Goldman-Rakic 1988). Recently, repeated scanning within individuals has delineated the networks with high spatial precision (Braga and Buckner 2017; Gordon et al. 2017; Laumann et al. 2015; see also Fedorenko et al. 2010, 2012; Huth et al. 2016; Michalka et al. 2015; Peer et al. 2015). Using such procedures, we discovered that the distributed transmodal network, often called the “default network” (DN; Andrews-Hanna et al. 2014; Buckner et al. 2008; Raichle 2015), likely comprises at least two separate parallel networks (Braga and Buckner 2017). The two networks are closely juxtaposed throughout the cortex and interdigitated in a way that suggests why features of the more detailed organization are lost in group-averaged data. The two networks were referred to as *network A* and *network B*. One important distinction between the networks is that *network A* is coupled with the posterior parahippocampal cortex (PHC), whereas *network B* is not, suggesting that they may be functionally specialized.

Since our observation of two separate networks, multiple studies of individuals have revealed related distinctions, but none with the same parallel structure distributed throughout the cortex (e.g., see the “Default A” and “Default B” networks in Fig. 4 of Kong et al. in press; the “Default” network in Fig. 3 of Gordon et al. 2017; see also Laumann et al. 2015; Wang et al. 2015). Thus the details of network organization require replication and further exploration. Of particular interest are zones of cortex that have been variably identified as “cores” or “hubs” of the DN. The designations reflect results that suggest the anterior and posterior midline are convergence zones of functional coupling between distributed systems (e.g., Buckner et al. 2009; Tomasi and Volkow 2010; see also Braga et al. 2013) and are active across multiple task classes (e.g., Andrews-Hanna et al. 2010, 2014) that otherwise show specificity (see also Power et al. 2013). The recent reevaluation of the functional anatomy within individuals necessitates a more detailed look at the anterior and posterior midline with higher spatial resolution methods that are able to disambiguate whether separate networks converge on common regions or, alternatively, whether separate functional regions lay side by side (Fedorenko et al. 2012; Michalka et al. 2015).

High-resolution imaging at high field offers a potential means to refine our understanding of network organization, in particular for regions that have complex organization including the anterior and posterior midline. Two prior studies have examined functional connectivity of the DN at high field. Hale et al. (2010) demonstrated that the DN could be identified at 7T with high signal-to-noise ratio, requiring lower spatial smoothing than conventional 3T imaging. Their proof-of-concept analysis focused on similarities between 3T and 7T group data. While robustly identifying the canonical DN, their analysis did not reveal any indications of substructure within the DN (which was not the focus of the study). De Martino et al. (2011) extensively analyzed the spatial properties of the DN within individuals at 7T and made two relevant observations. First, the DN could be resolved within individuals using high-resolution acquisition (1–1.5 mm) and minimal spatial smoothing. Second, they found that the DN regions were largely located within the gray matter, often following the cortical ribbon. Their analyses focused on the DN as defined in group-averaged data, and therefore they did not seek or detect spatially juxtaposed networks within the canonical DN. Nonetheless, the observation that the regions followed the cortical gray matter ribbon bodes well for the use of high-field functional magnetic resonance imaging (fMRI) to resolve spatial details of functional connectivity networks within individuals. Of particular interest, Peer et al. (2015) examined task-based responses in or near regions associated with the DN at high resolution. They observed a complex organization that included both domain-general as well as domain-specific regions along the posterior midline.

In the present study, we acquired new data to explore further the hypothesis that there exist parallel interdigitated networks within the canonical group-defined DN. Critically, in addition to 3T fMRI data that could replicate our earlier analyses, we also explored high-resolution fMRI data acquired over multiple runs at 7T to delineate the parallel networks in relation to the topography of the cortical ribbon itself. Closely juxtaposed and often minimally overlapping portions of the two networks were identified along the cortical ribbon throughout the brain, including within midline locations previously considered zones of convergence.

## METHODS

### Participants

Five healthy right-handed adult women were recruited from the greater Boston community and screened to exclude a history of neurological and psychiatric illness. Data were collected as part of two separate studies. The 3T study acquired fMRI data using procedures similar to those of Braga and Buckner (2017). The 7T study acquired data at higher spatial resolution. Participants provided written informed consent, and procedures were approved by the Institutional Review Board of Harvard University (3T study) and Partners Healthcare (7T study). 3T study participants were each scanned across 31 separate MRI sessions (*n* = 2; ages 22 and 23 yr; over 28 wk for one individual and 40 wk for the other). 7T study participants were each scanned in a single MRI session (*n* = 3; ages 19–28 yr).

### 3T MRI Data Acquisition

Data were acquired at the Harvard Center for Brain Science on a Siemens Prisma-fit 3T MRI scanner using the vendor’s 64-channel phased-array head-neck coil (Siemens, Erlangen, Germany). The vendor’s head-coil-shaped pillow was used with additional eggshell foam padding on the top and side of the head for immobilization. The scanner room was illuminated to enhance alertness. Eyes were monitored and video-recorded using an Eyelink 1000 Core Plus with Long-Range Mount (SR Research, Ottawa, ON, Canada). A four-point scale was used to record the participant’s level of arousal during each run based on the frequency and duration of eye closures. A video of the eye tracker output (showing eye closures) was also retained to assess and quantify compliance.

Each subject’s 31 MRI sessions included at least two blood oxygenation level-dependent (BOLD) runs of fixation, each lasting 7 min 2 s. Participants were instructed to remain still, stay awake, and maintain fixation on a centrally presented black crosshair viewed on a light gray background through a mirror attached to the head coil. The screen was adjusted to ensure comfortable viewing. Sessions included additional task runs (“n-back” working memory and visuomotor tasks) and an arterial spin labeling sequence that are not analyzed here.

Functional runs were flagged for exclusion if *1*) maximum absolute motion exceeded 2 mm, *2*) slice-based temporal signal-to-noise ratio was ≤135, or *3*) the value for maximum absolute motion or signal-to-noise ratio represented an outlier when values from all runs were plotted together. The raw data from flagged runs were then visually checked for motion artifacts and excluded if these were deemed to be severe. Following this procedure, no runs were excluded for *subject 1* (S1) and one run was excluded for S2. Because of data loss (failures) during processing, 3 additional runs were discarded for S1 and 9 additional runs were discarded for S2, leaving a total of 59 and 53 runs, respectively.

BOLD fMRI (Kwong et al. 1992; Ogawa et al. 1992) data were acquired using a multiband gradient-echo echo-planar pulse sequence (Setsompop et al. 2012): TR = 1,000 ms, TE = 32.6 ms, flip angle = 64°, 2.4-mm isotropic voxels, matrix 88 × 88 × 65, multislice 5× acceleration. The custom sequence was generously provided by the Center for Magnetic Resonance Research at the University of Minnesota. Minimization of signal dropout was achieved by automatically (van der Kouwe et al. 2005) selecting a slice plane 25° from the anterior-posterior commissural plane toward the coronal plane (Mennes et al. 2014; Weiskopf et al. 2006). A rapid T1-weighted anatomical scan was acquired in each session using a multiecho magnetization-prepared rapid gradient echo (MPRAGE) three-dimensional sequence (van der Kouwe et al. 2008): TR = 2,200 ms, TE = 1.57, 3.39, 5.21, 7.03 ms, TI = 1,100 ms, flip angle = 7°, 1.2-mm isotropic voxels, matrix 192 × 192 × 144, in-plane generalized autocalibrating partial parallel acquisition (GRAPPA) acceleration 4 (see Nielsen et al. 2019 for analysis of this rapid variant). A dual gradient-echo B0 field map was acquired to correct for susceptibility-induced gradient inhomogeneities: TE = 4.45, 6.91 ms with slice prescription/spatial resolution matched to the BOLD sequence.

The broader scope of the 3T data (which extended beyond the present aims) was to assess the effects of transcranial magnetic stimulation (TMS) on functional connectivity in individuals (stimulation administered before the MRI sessions). In our hands, the effects of TMS on spatial topography of cortical organization are not detectable, so the data were treated as standard rest fixation for the present purposes (confirming this decision, the present findings did not differ when only data recorded after sham stimulation were examined).

For seed-based functional connectivity analyses in both the volume and surface, the 3T data were divided into four independent data sets (*n* = 12 in each data set, yielding 1 h 22 min of data in each data set after exclusion of the initial 12 volumes in each run for T1 equilibration effects). These multiple data sets were used to replicate the observed network distinctions. Best-estimate seed-based functional connectivity maps were created by averaging the maps produced from the four data sets in each subject. For *k*-means clustering in both the volume and surface, 59 runs were simultaneously included for S1 (yielding 6 h 43 min 10 s of usable data) and 53 runs for S2 (6h 02m 10s of usable data).

### 7T MRI Data Acquisition

Data were acquired at the Athinoula A. Martinos Center for Biomedical Imaging on a whole-body 7T MRI scanner (Siemens, Erlangen, Germany) equipped with SC72 body gradients (70 T/m maximum gradient strength and 200 T·m^−1^·s^−1^ maximum slew rate) and a 32-channel radiofrequency loop coil head array (Keil et al. 2010) for reception and detunable bandpass birdcage coil for transmission. A pillow was used for head comfort and immobilization. The scanner room was dimly lit. Participants were instructed to lie as still as possible with their eyes open for the duration of the run. Four eyes-open “resting state” runs were acquired in a single MR session from each participant (each 6 min 5 s, yielding ~24 min 14 s of data after removal for T1 equilibration effects). One run was discarded for S2 due to motion yielding 18 min 10 s of data. A motor task was also collected but is not analyzed here.

Functional imaging data were acquired using accelerated, multiband single-shot gradient-echo echo-planar imaging, with robust autocalibration using the fast low-angle excitation echo-planar technique (FLEET-ACS; Polimeni et al. 2016) sensitive to BOLD contrast: TR = 1,490 ms, TE = 24.0 ms, flip angle = 60°, 1.35-mm isotropic voxels, matrix 142 × 142 × 81, multislice 3× acceleration, GRAPPA factor 3. The slice acquisition plane was tilted toward the coronal plane until coverage of the cerebellum was achieved. A T1-weighted anatomical scan was acquired for each participant using multiecho MPRAGE: TR = 2,530 ms, TE = 1.76, 3.7 ms, TI = 1,100 ms, flip angle = 7°, 0.75-mm isotropic voxels, matrix 320 × 320 × 224, GRAPPA factor 2.

### 3T Data Processing

A custom in-house preprocessing pipeline (“iProc”) optimized within-subject data alignment across different scanning sessions, preserving anatomical detail as much as possible by minimizing spatial blurring and multiple interpolations (expanding on Braga and Buckner 2017; Poldrack et al. 2015; Yeo et al. 2011). Each subject’s data were processed separately. To optimize alignment, two subject-specific registration templates were created: a mean BOLD template and a T1 native-space template (as described below). Spatial alignment was achieved by calculating five transformation matrices to be applied to each usable BOLD volume. For each BOLD volume, three transforms were calculated to *1*) correct for head motion, *2*) correct for geometric distortions caused by susceptibility gradients (using the acquired B0 field map), and *3*) register the BOLD volume to the within-subject mean BOLD template. Two further transforms were calculated once for each subject and applied to all registered volumes. These transforms projected data *4*) from the mean BOLD template to the T1 native-space template and *5*) from the T1 native-space template to the MNI ICBM 152 1-mm atlas (Mazziotta et al. 1995). The transformation matrices were composed into a single matrix that was applied to the original BOLD volumes in a single interpolation to reduce spatial blur. BOLD data were projected to native space by composing *matrices 1–4* and to MNI space by composing *matrices 1–5*. Registration details are described below. Data were also projected to the cortical surface and prepared for functional connectivity as detailed below.

#### Within-subject spatial alignment.

Motion correction was achieved by aligning all volumes in a run to the middle volume of that run (*matrix 1*; linear registration using MCFLIRT, FSL v5.0.4; Jenkinson et al. 2002). Distortion correction of the middle volume was achieved using the field map collected in that same MR session, yielding a nonlinear transformation matrix (*matrix 2*; calculated using FSL FUGUE; FSL v4.0.3; Jenkinson 2004). Visual inspection of each session’s field map showed that inhomogeneities varied from session to session, hence a session-specific field map was used to optimize distortion correction.

The subject-specific mean BOLD template was created iteratively. First, the distortion-corrected middle volume of the run acquired immediately before the field map in the subject’s first MR session was selected as an interim registration target. The interim target was upsampled to 1.2-mm isotropic space to aid subsequent spatial alignment of BOLD volumes (using FLIRT, FSL v5.0.4; Jenkinson and Smith 2001). Next, all of the distortion-corrected middle volumes from each run were registered to this interim target using linear registration at 12 degrees of freedom (dof; using FLIRT, FSL v5.0.4; Jenkinson and Smith 2001). The aligned BOLD images were averaged, creating the within-subject mean BOLD template in 1.2-mm isotropic space. In this way, BOLD data from every run contributed to the subject’s mean BOLD template, minimizing bias toward any one run or session.

**Fig. 1.**
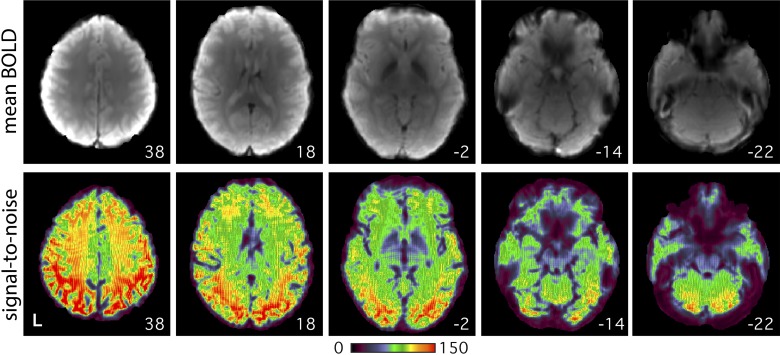
Images show the quality of the 3T data. Axial slices from example *subject 1* (S1; 3T study) display the mean blood oxygenation level-dependent (BOLD) image (*top*) and temporal signal-to-noise ratio map (*bottom*). High signal-to-noise ratio was obtained throughout much of the brain. Ventral regions, particularly along the medial and lateral temporal lobe and prefrontal cortex, suffered from decreased signal and unrecovered spatial distortion. Note these data have been corrected for spatial distortions due to susceptibility gradients. Numbers correspond to MNI coordinates for each slice. Left hemisphere is on the *left* of each axial slice.

Alignment between the distortion-corrected middle volume of each run and the subject-specific mean BOLD template was achieved using linear registration at 12 dof (*matrix 3*; FLIRT, FSL v5.0.4; Jenkinson and Smith 2001). The T1 native-space template was created by selecting a T1-weighted structural image (upsampled to 1-mm isotropic space) that was visually deemed to have good pial and white matter boundary surface estimates as calculated automatically by FreeSurfer’s recon-all (Fischl et al. 1999). Projection of data from the mean BOLD template to T1 native space was achieved using linear registration at 12 dof (*matrix 4*; FLIRT, FSL v5.0.4). Projection of data from T1 native space to MNI space was performed using nonlinear registration (*matrix 5*; FNIRT, FSL v5.0.4; Andersson et al. 2007). A mean temporal signal-to-noise-ratio map (Fig. 1) was created in MNI space by dividing the mean by the standard deviation of the BOLD signal (voxelwise) within each run and then averaging across all runs in each subject.

#### Preprocessing for functional connectivity.

Nuisance variables (6 motion parameters plus whole brain, ventricular, and deep white matter signal) and their temporal derivatives were calculated from BOLD data in T1 native space. The signals were regressed out of both native- and MNI-space data (using 3dTproject, AFNI v2016.09.04.1341; Cox 1996, 2012). This was followed by bandpass filtering at 0.01–0.1 Hz (using 3dBandpass, AFNI v2016.09.04.1341; Cox 1996, 2012). MNI-space data were smoothed using a 2-mm full width at half-maximum (FWHM) kernel (using fslmaths, FSL v5.0.4; Smith et al. 2004). For surface analysis, data were resampled from the native space to the fsaverage6 standardized cortical surface mesh (40,962 vertices per hemisphere; Fischl et al. 1999) and then surface-smoothed using a 2-mm FWHM kernel. Data were sampled from the gray matter halfway between the white and pial surfaces using trilinear interpolation.

The iProc pipeline thus allowed for high-resolution and robustly aligned BOLD data, with minimal interpolation and signal loss, output to three final spaces: the native space, MNI space, and the fsaverage6 cortical surface. The use of the MNI and fsaverage common reference spaces allowed us to display the data using standardized orientation, coordinates, and location of gross anatomical landmarks while preserving each individual’s idiosyncratic anatomy. Data were checked during matrix calculations for registration errors and quality control. Note that, unconventionally, BOLD data were upsampled to 1-mm isotropic space (in both native and MNI spaces). This substantially increased the computational load of all preprocessing and analysis steps, but was found in preliminary analyses to produce functional connectivity maps with noticeably better resolved regions displaying finer anatomical details that were largely confined to the gray matter. The improvement was visible even compared with data that were more modestly upsampled to 1.5-mm isotropic space.

### 7T Data Processing

The 7T data were preprocessed using similar procedures to the 3T data with a few key differences. Notably, no field maps were available, so no distortion correction was applied. A subject-specific mean BOLD alignment target was created by *1*) calculating the mean BOLD image for each run, before removal of the first four volumes and motion correction, *2*) registering the mean images to MNI ICBM 152 1-mm space (Mazziotta et al. 1995) using linear registration (FLIRT, FSL v5.0.4; Jenkinson and Smith 2001), and then *3*) calculating the mean of the registered images. Functional data were then processed as follows: *1*) The initial four volumes of each run were discarded to allow for T1-equilibration effects. *2*) Head motion correction transforms (*matrix 1*) were calculated by registering each volume to the mean BOLD image from each run (calculated after alignment to the middle volume of each run; using MCFLIRT, FSL v5.0.4; Jenkinson et al. 2002). *3*) Transforms were calculated for registration between each run’s mean BOLD image and the subject-specific mean BOLD template (*matrix 2*) using nonlinear registration (FNIRT, FSL v5.0.4; Andersson et al. 2007). *4*) Transformation *matrices 1* and *2* were composed into a single matrix that was applied to each original BOLD volume so that motion correction and registration to the mean BOLD template could be achieved in a single interpolation step to reduce spatial blur. *5*) Nuisance variables (6 motion parameters plus signals from whole brain as well as lateral ventricles and deep white matter, as hand-drawn for each subject) and their temporal derivatives were regressed out (using fsl_regfilt, FSL v5.0.4). *6*) Data were bandpass filtered between 0.01 and 0.1 Hz and smoothed (using 3dBandpass, AFNI v2016.09.04.1341; Cox 1996). Two subjects (S1 and S3) were smoothed at 2.5-mm FWHM, whereas the other (S2) was smoothed at 3.0-mm FWHM, because these kernels produced robust functional connectivity maps with minimal smooth in preliminary analyses using kernels ranging from 2- to 4-mm FWHM. A mean temporal signal-to-noise-ratio map (see Fig. 5) was calculated for each subject by dividing the mean by the standard deviation voxelwise within each BOLD run, prior to filtering and smoothing, and then averaging across all runs.

### Functional Connectivity Analysis

For the 3T data, functional connectivity analyses were performed using both seed-based and data-driven parcellation techniques both in the volume and on the surface. For the 7T data, a seed-based technique was used in the volume.

#### 3T surface-based analysis.

Pearson’s product-moment correlations between the fMRI timeseries at each vertex were computed, yielding an 81,924 × 81,924 correlation matrix (40,962 vertices per hemisphere) for each run of BOLD data. These matrices were Fisher-transformed and averaged together, yielding a within-subject across-run mean correlation matrix with high stability. This average matrix was then inverse Fisher-transformed back to correlation values and assigned to the vertices of a cortical template created in-house by combining the left and right hemispheres of the fsaverage6 surface into the CIFTI format (as described in Braga and Buckner 2017). This allowed individual vertices to be selected and the resulting correlation maps to be visualized rapidly using the Connectome Workbench’s wb_view software (Marcus et al. 2011).

For seed-based analysis in each subject, individual candidate seed vertices were manually selected to target *networks A* and *B*. The seed selection process was constrained to the left dorsolateral prefrontal cortex, because this region was found to contain distinct representations of both networks by Braga and Buckner (2017). Furthermore, because in a functional connectivity map the correlation values near to the seed are inflated due to factors such as blurring, the selection of seeds in this region allowed the detailed topography of the two networks to be better defined in distal regions (e.g., along midline and temporal cortices), which more characteristically distinguish the two networks (see anatomical description of network features in results). Trial and error was used to home in on seed vertices that showed connectivity patterns resembling *networks A* and *B* by eye, by comparison with the networks as defined previously (Braga and Buckner 2017). Through this iterative process, the correlation pattern revealed by each seed was used to inform successive seed selections. A seed vertex was deemed a suitable candidate for *network A* or *B* when its resulting correlation pattern had high values and a spatial distribution that was representative of one of the networks. Specifically, the precise locations of regions were examined in different cortical zones, along the posterior and anterior midline, the lateral temporal cortex, and the medial temporal lobe (MTL), where the two networks were easily differentiated.

Candidate seed vertices were recorded (using each vertex’s unique number), and once a pool of 6–10 candidate vertices had been obtained, the maps from each candidate were compared with each other by eye, and the most robust map was chosen to be representative for each network. A network was deemed robust if the correlation values were high, but also if the network regions displayed sharp boundaries. In other words, if the network regions (i.e., areas showing high correlation values) were surrounded by broad areas containing lower correlation values, this was taken as evidence that the seed vertex contained some mixture of signals, leading to nonspecific correlations. On the other hand, if the network regions had sharp cutoffs and were surrounded by areas of low correlations, this was taken as evidence that the seed was sampling a single network (at the level of resolution available here). By this process, a single seed location was selected for *networks A* and *B* for each subject. For final visualization of seed-based connectivity maps, correlation values were converted back to *z*(*r*) using the Fisher transform.

For automated parcellation analysis in each subject, *k*-means clustering was implemented that was independent of experimenter interaction (and hence potential bias). Preprocessed data were concatenated in time, and MATLAB’s *kmeans* function (v2015b; The MathWorks, Natick, MA) was used to parcellate the timeseries into 12 clusters on the surface (and also in MNI space). Default settings (1 random initialization, 100 iterations) were used. As the results will reveal, highly similar network estimates were found for both the seed-based and parcellation approaches, suggesting that the discoveries are robust to the exact network discovery method applied.

#### 3T volume-based analysis.

MNI-space data were analyzed using AFNI’s InstaCorr (Cox 1996, 2012; Cox and Saad 2010). 3dSetUpGroupInCorr calculated the Pearson’s product-moment correlation coefficients between all pairs of voxels within a brain mask for each BOLD run. The brain mask was used to exclude nonbrain voxels to reduce the computational load. The matrices were Fisher-transformed before averaging across runs using 3dGroupInCorr. This platform allows interactive selection of individual voxels and rapid visualization of their seed-based correlation maps.

The surface-defined seed vertices selected to target *networks A* and *B* (above) were used to identify the seed voxels in the volume. To obtain MNI coordinates corresponding to the surface seed vertices, the position of each *x*-, *y*-, and *z*-slice in MNI space was encoded with an ascending integer, creating three reference volumes. These reference volumes were projected to the fsaverage6 surface by first applying the inverse of each subject’s native-space-to-MNI-space transformation matrix and then projecting the reference volumes to the surface using the same sampling procedure used in data preprocessing (mri_vol2surf, FreeSurfer; Fischl et al. 1999). The exception was that both these transformations were performed using nearest neighbor interpolation to maintain the *x*-, *y*-, and *z*-axis coordinates as integers. This produced three surface maps where each vertex contained a value corresponding to that vertex’s location along *x*-, *y*-, or *z*-axes in MNI space. In this way, the MNI coordinates corresponding to the selected surface vertices for *networks A* and *B* were used to define the volume maps.

#### 7T volume-based analysis.

Native-space data were analyzed using AFNI’s InstaCorr as described above. The within-subject mean BOLD template was used as an underlay for anatomical guidance. At this resolution (data acquired at 1.35 mm and upsampled to 1-mm isotropic space), the gyral and sulcal anatomy could be observed in the mean BOLD template, providing anatomical detail with the same distortion profile as the actual BOLD data used to calculate functional connectivity. Anatomical landmarks were identified by comparison with a reference atlas containing brain images and anatomical labels at different slices and orientations (Duvernoy 1999). Individual seed voxels were selected from the gray matter along the dorsolateral prefrontal cortex of each subject at or near the superior frontal sulcus and superior frontal gyrus, and the resulting correlation maps were visualized at a threshold of *z*(*r*) > 0.2. The seed voxels yielding the best estimates of the two networks were determined by checking the anatomical locations of regions in different cortical zones, along the posterior and anterior midline, the MTL (for *network A*), and the inferior frontal gyrus (for *network B*), where the two networks were easily differentiated (see description of network features in results).

#### Overlap maps.

To visualize the spatial relationships between the two networks, overlap maps were created by thresholding the correlation maps at *z*(*r*) > 0.2 and binarizing. The binarized *network B* map was multiplied by a scalar value of 2, and the binarized *network A* map was subtracted from the scaled *network B* map. This resulted in an image where *network A*-specific voxels had a value of −1, *network B*-specific voxels had a value of +2, and the overlap voxels had a value of +1. The overlap maps were visualized in wb_view (Marcus et al. 2011) using the FSL color bar set to range from −5.0 to 0 and +0.5 to +2.5.

### Experimental Design and Statistical Analysis

This study includes five participants, two scanned over 31 fMRI sessions at 3T and three scanned in 1 session at 7T. Data were averaged over four independent data sets, each including 12 fMRI runs to test the reliability of the connectivity patterns in the 3T study. The large volume of data allowed three independent replications within each subject. Data were averaged over 59 and 53 fMRI runs for data-driven clustering and averaged over the four replication data sets (*n* = 48) for seed-based best estimates of connectivity patterns in the 3T study. Data were averaged over three and four fMRI runs in the 7T study (see details in *Functional Connectivity Analysis*). Functional connectivity between brain regions was calculated in MATLAB (version 2015b; https://www.mathworks.com) using Pearson’s product-moment correlations and Fisher’s *r*-to-*z* transformation before averaging across runs. Network parcellation was performed using MATLAB’s *kmeans* function (version R2015b).

## RESULTS

### Parallel Distributed Networks Are Identified Within Individuals

**Fig. 2.**
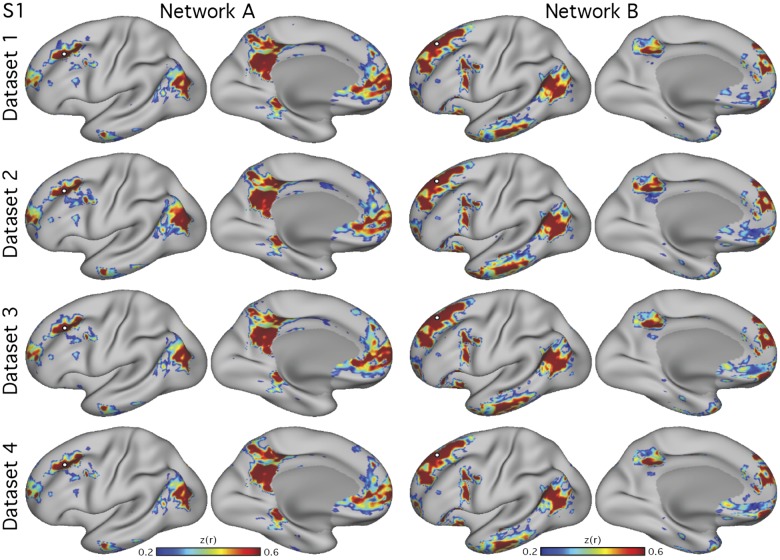
Parallel closely juxtaposed distributed networks are reliable in *subject 1* (S1). Each column shows the functional connectivity maps from a single seed vertex (white circle) selected from the left dorsolateral prefrontal cortex of S1 (3T study). The *left* pair of columns shows *network A*, and the right pair shows *network B*. Data from S1 were divided into 4 independent data sets. The two networks, *network A* and *network B*, were reliably observed in all 4 data sets. Each seed pair produced distinct distributed networks that occupied closely juxtaposed regions within zones typically considered part of the canonical “default network.” Lateral and medial inflated views of the left hemisphere are shown. The surfaces are rotated by 19° along the *y*-plane to better show the ventromedial prefrontal cortex. *z*(*r*), Fisher’s *r*-to-*z* transformed Pearson’s product-moment correlations.

**Fig. 3.**
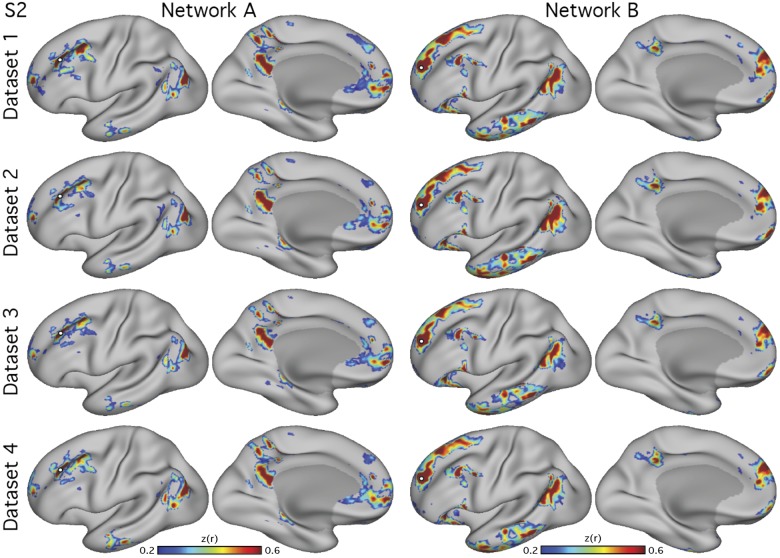
Parallel closely juxtaposed distributed networks are reliable in *subject 2* (S2). Analysis of another individual, S2 (3T study), recapitulated the observation of the two parallel interdigitated networks, *network A* and *network B*, consistently across 4 data sets. Each column shows the functional connectivity maps from a single seed vertex (white circle) selected from the left dorsolateral prefrontal cortex of S2. Lateral and medial inflated views of the left hemisphere are shown. *z*(*r*), Fisher’s *r*-to-*z* transformed Pearson’s product-moment correlations.

*Networks A* and *B* within the canonical group-averaged DN could be identified in all five individuals (Figs. 2, 3, and 6–8). In each individual, the networks recapitulated previously described features, in particular the distributed nature of both networks, the closely juxtaposed regions observable in multiple zones of the cortex, and the close interdigitated arrangement of the two networks (Braga and Buckner 2017). Specifically, *networks A* and *B* are distributed within canonical DN regions in the posterior medial cortex including posterior cingulate (PCC) and retrosplenial cortex (RSC), the anterior medial cortex extending throughout medial prefrontal cortex (MPFC) including anterior cingulate cortex (ACC), the inferior parietal lobule (IPL) extending rostrally to the temporoparietal junction (TPJ), and the lateral temporal cortex (extending the length of the superior temporal sulcus and middle temporal gyrus).

There were multiple anatomical details that differed between the networks. The two most prominent differences, perhaps to be considered diagnostic features that can be used heuristically to distinguish the networks, are *1*) *network A* shows strong connectivity with a region of posterior PHC, whereas *network B* does not, and *2*) *network B* shows connectivity with rostral regions of the IPL extending into TPJ, whereas *network A* generally shows connectivity with more caudal regions of the IPL.

The relative positions of the two networks within the many canonical DN regions revealed further reliable differences, including *3*) *network A* occupies more ventral regions of the posterior midline, including those at or adjacent to the RSC and ventral PCC, whereas *network B* is typically located in a more focal dorsal region of the PCC, sparing the RSC, *4*) *network B* involves a small circumscribed region of the ventromedial prefrontal cortex that sits beneath the regions showing connectivity with *network A*, *5*) *network B* occupies a large swath of lateral temporal cortex that extends along the majority of the middle temporal gyrus and includes the temporal pole, whereas *network A* occupies smaller circumscribed regions in rostral lateral temporal cortex, and *6*) *network B* contains a large region covering multiple gyri along the lateral inferior frontal cortex, whereas *network A* contains only a small region more dorsally near the inferior frontal sulcus. These details replicated across the four independent data sets in both 3T study subjects (Figs. 2 and 3) and in the three 7T study subjects (Figs. 6–8), building confidence in the separation between *networks A* and *B*. A further analysis confirmed that the effect of TMS stimulation on the topography of functional connectivity patterns was negligible (not shown).

### Networks Are Observed Using Multiple Analysis Approaches

**Fig. 4.**
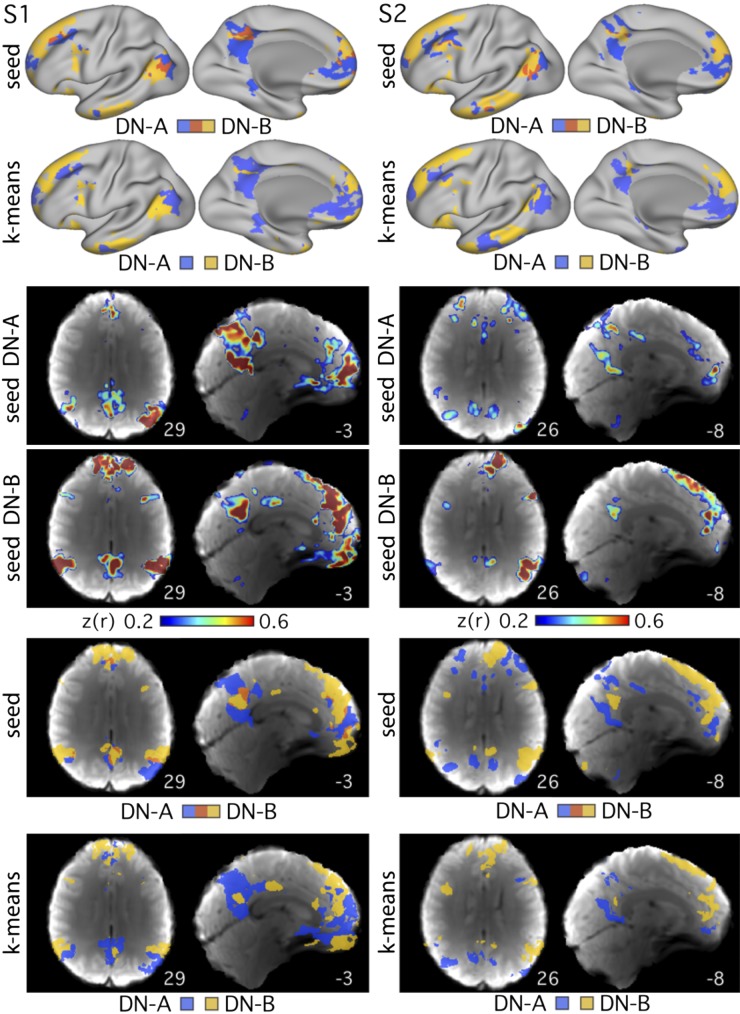
Networks are reproduced using multiple analysis methods. Data are shown on the surface (*top* 2 rows) and volume (remaining rows) for the 3T study. Each column shows maps produced using all data from *subject 1* (S1; *left* columns) or *subject 2* (S2; *right* columns). The two networks, *network A* (DN-A; in blue) and *network B* (DN-B; in yellow), were defined using seed-based correlation and data-driven parcellation (*k*-means clustering at *k* = 12). The “seed DN-A” and “seed DN-B” rows show the individual seed-based correlation maps from seeds in the dorsolateral prefrontal cortex. The overlap images (“seed” rows) show the same seed-based connectivity maps thresholded at *z*(*r*) > 0.4 for S1 and *z*(*r*) > 0.25 for S2, and then binarized, with regions of overlap displayed in red. Numbers correspond to MNI coordinates for each slice. Left hemisphere is on the *left* of each axial slice.

A potential concern was that the manual seed-selection process may have introduced observer bias toward correlation patterns that confirm *network A* and *network B*. To address this concern, we performed data-driven *k*-means clustering to parcellate the timeseries from all surface vertices and gray matter voxels into discrete networks. The *k*-means images in Fig. 4 show that data-driven clustering also delineated both *networks A* and *B* in both subjects (S1 and S2). This confirms that the observation of the two networks generalizes across analysis methods and is not dependent on observer input.

Another possible concern surrounds distortions that might arise from surface projection. Specifically, the surface sampling procedure can induce breaks in the spatial pattern of activations by splitting a single contiguous region in the volume into multiple regions on the surface. An interdigitation pattern might thus arise artificially as a consequence. Figure 4 shows the two networks, *network A* and *network B*, and their overlap, defined on the surface and in the volume. The two networks occupy closely juxtaposed regions throughout the brain in the volume and show notable interdigitation of regions particularly along the posterior and anterior midline, indicating that the interdigitation was not contingent on the surface projection step. Similar features were also observed in the volume in the higher resolution data (see Figs. 6–8). The replication and generalized observation of distinct *networks A* and *B* through multiple forms of data acquisition, preprocessing, and analysis techniques provide strong evidence that they represent real features of functional organization.

### Closely Juxtaposed Networks Follow the Cortical Ribbon at High Resolution

**Fig. 5.**
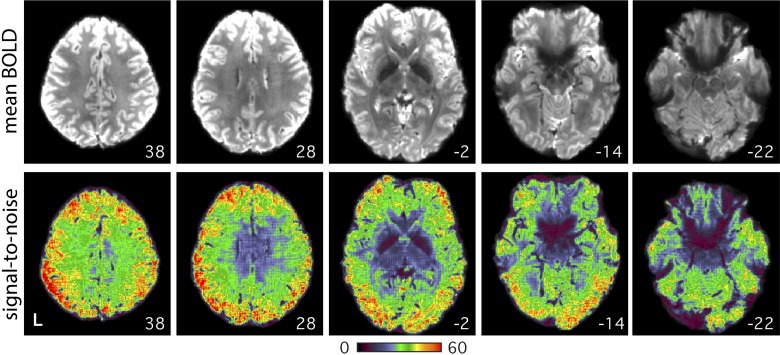
Images show the quality of the high-resolution 7T data. Axial slices from example *subject 3* (S3; 7T study) display the mean blood oxygenation level-dependent (BOLD) image (*top*) and temporal signal-to-noise ratio map (*bottom*). Numbers correspond to MNI coordinates for each slice. Left hemisphere is on the *left* of each axial slice. Note the 7T data were not corrected for spatial distortions due to susceptibility gradients.

**Fig. 6.**
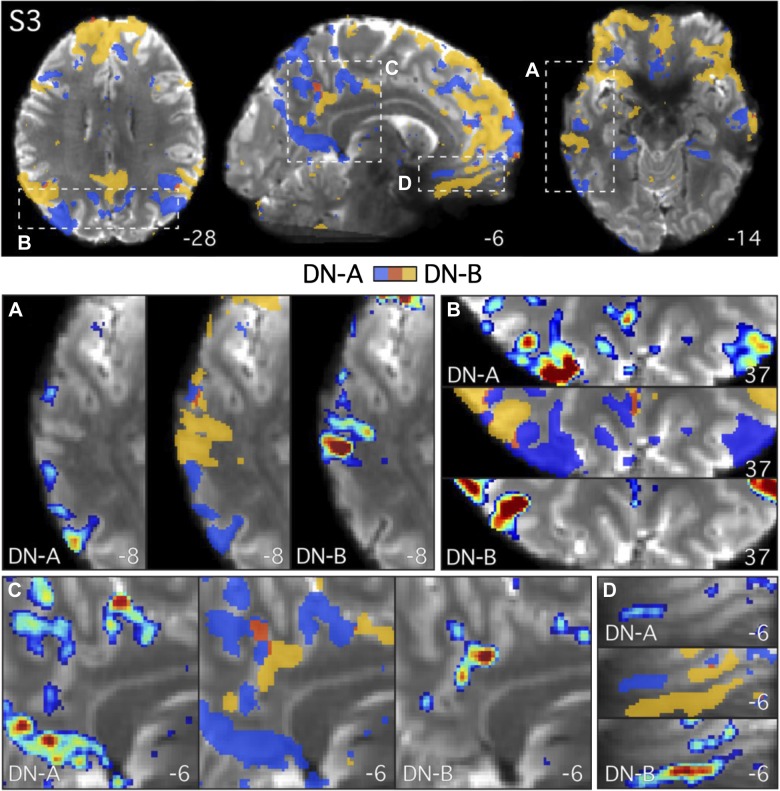
Parallel closely juxtaposed distributed networks revealed at high resolution in *subject 3* (S3). Both *network A* (DN-A; in blue) and *network B* (DN-B; in yellow) were defined in S3 (7T study) at high resolution using seed-based functional connectivity. Individual seed voxels were selected from the left dorsolateral prefrontal cortex (seeds not shown). Functional connectivity maps are thresholded at *z*(*r*) > 0.2 in all images and binarized in the overlap images to show both networks and their overlap (in red). Note that even at this liberal threshold, minimal overlap is observed. The subject’s mean blood oxygenation level-dependent image is displayed as the underlay to allow the gray matter (lighter gray) and white matter (darker gray) anatomy and cerebrospinal fluid (white) to be seen. The regions closely follow the curvature of the gray matter and are interdigitated in complex ways along the cortical ribbon. *Top*: full axial and sagittal slices show the distributed parallel organization of the two networks. *A–D*: zoomed-in images of the lateral temporal cortex (*A*), the parietal lobes (*B*), and the posterior (*C*) and ventral anterior midline (*D*) highlight the close juxtaposition of the regions. Functional connectivity maps are displayed using the color bar range (0.2–0.6) as shown in Fig. 4. Numbers correspond to MNI coordinates for each slice. Note that different slices are sometimes shown in *A*–*D* and *top* panel. Left hemisphere is on the *left* of each axial and coronal slice.

**Fig. 7.**
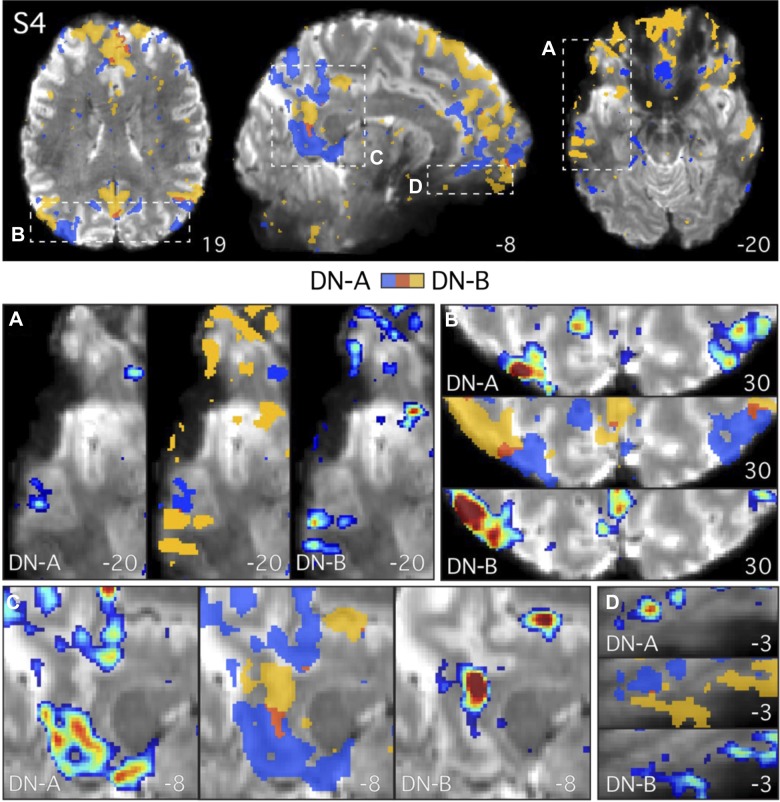
Parallel closely juxtaposed distributed networks revealed at high resolution in *subject 4* (S4). Analysis of S4 (7T study) recapitulated features observed in S3 (see Fig. 6). The two networks, *network A* (DN-A; in blue) and *network B* (DN-B; in yellow), tightly follow the gray matter anatomy and are closely juxtaposed in multiple cortical zones. Individual seed voxels were selected from the left dorsolateral prefrontal cortex (seeds not shown). Functional connectivity maps are thresholded at *z*(*r*) > 0.2 in all images and binarized in the overlap images to show both networks and their overlap (in red). The subject’s mean blood oxygenation level-dependent image is displayed as the underlay to allow the gray matter (lighter gray) and white matter (darker gray) anatomy and cerebrospinal fluid (white) to be seen. *Top*: full axial and sagittal slices show the distributed parallel organization of the two networks. *A–D*: zoomed-in images of the lateral temporal cortex (*A*), the parietal lobes (*B*), and the posterior (*C*) and ventral anterior midline (*D*) highlight the close juxtaposition of the regions. Functional connectivity maps are displayed using the color bar range (0.2–0.6) as shown in Fig. 4. Numbers correspond to MNI coordinates for each slice. Note that different slices are sometimes shown in *A*–*D* and *top* panel. Left hemisphere is on the *left* of each axial and coronal slice.

**Fig. 8.**
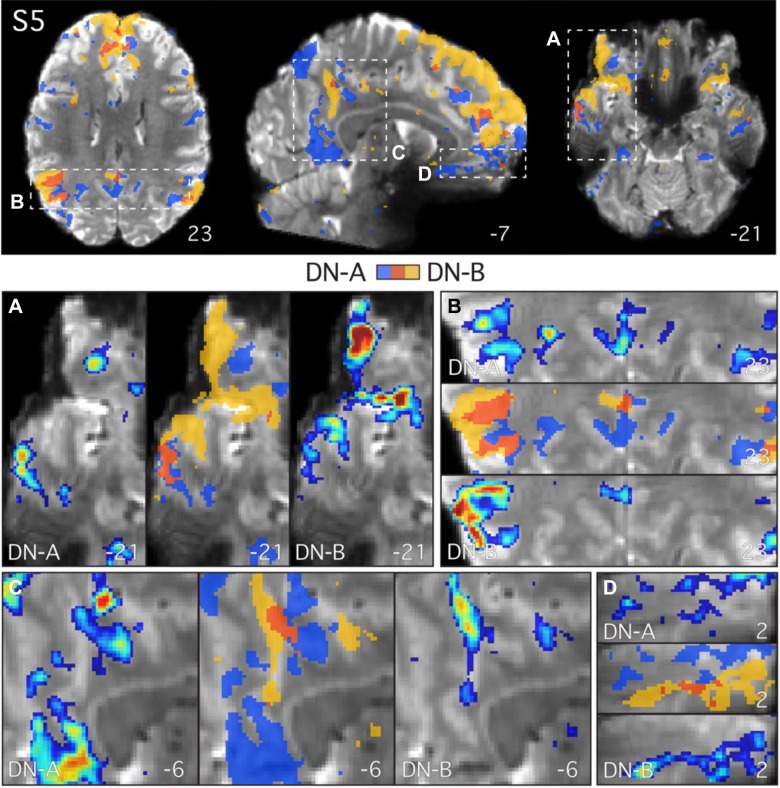
Parallel closely juxtaposed distributed networks revealed at high resolution in *subject 5* (S5). Analysis of S5 (7T study) recapitulated detailed features observed in S3 (Fig. 6) and S4 (Fig. 7). The two networks, *network A* (DN-A) and *network B* (DN-B), again follow the gray matter anatomy and are closely juxtaposed in multiple cortical zones. This individual displays more overlap than the others at this threshold. Individual seed voxels were selected from the left dorsolateral prefrontal cortex (seeds not shown). Functional connectivity maps are thresholded at *z*(*r*) > 0.2 in all images and binarized in the overlap images to show both networks and their overlap (in red). The subject’s mean blood oxygenation level-dependent image is displayed as the underlay to allow the gray matter (lighter gray) and white matter (darker gray) anatomy and cerebrospinal fluid (white) to be seen. *Top*: full axial and sagittal slices show the distributed parallel organization of the two networks. *A–D*: zoomed-in images of the lateral temporal cortex (*A*), the parietal lobes (*B*), and the posterior (*C*) and ventral anterior midline (*D*) highlight the close juxtaposition of the regions. Functional connectivity maps are displayed using the color bar range (0.2–0.6) as shown in Fig. 4. Numbers correspond to MNI coordinates for each slice. Note that different slices are sometimes shown in *A*–*D* and *top* panel. Left hemisphere is on the *left* of each axial and coronal slice.

**Fig. 9.**
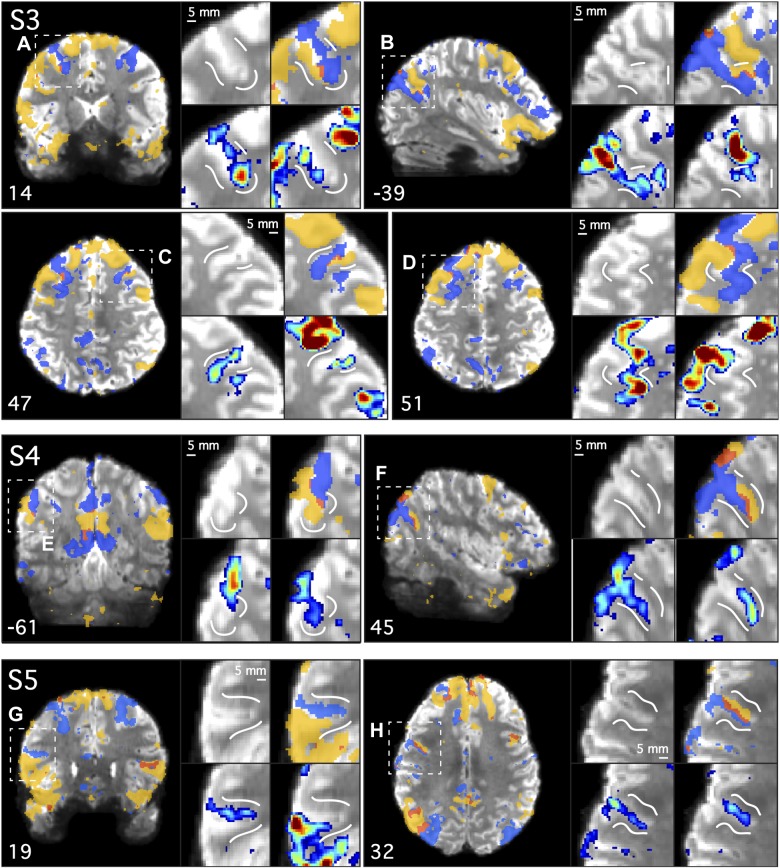
Networks are closely juxtaposed inside individual sulci. High-resolution data from 3 subjects are shown (S3, S4, and S5; 7T study). Selected slices illustrate locations where the two distributed networks fall in distinct regions of the same sulcus or junction between sulci. *Network A* (DN-A; in blue), *network B* (DN-B; in yellow), and their overlap (in red) are shown. In each panel, an overlap image is shown in the full coronal (*A*, *E*, *G*), sagittal (*B*, *F*), or axial slice (*C*, *D*, *H*), followed by 4 *insets* that zoom in on the dashed box to highlight anatomical details. *Insets* include each subject’s mean blood oxygenation level-dependent underlay for anatomical guidance (*top left*), the overlap image (*top right*), and the individual functional connectivity maps for *network A* (*bottom left*) and *network B* (*bottom right*). The white lines provide landmarks so that the relative position of each network can be considered across *insets*. The two networks were often observed on opposite banks of the same sulcus, deep into the fundus (see *A*, *C*, *G*, and *H*). Maps are thresholded at *z*(*r*) > 0.2 in all images, and functional connectivity maps are displayed using the color bar range (0.2–0.6) as shown in Fig. 4. Note other views of these same functional connectivity maps are displayed in Figs. 6–8.

The 7T data (Fig. 5) allowed the detailed anatomy of the distributed networks to be resolved at high resolution. Figures 6–9 zoom in on different zones of the DN so that the locations of the regions along the cortical ribbon can be appreciated. The two networks closely followed the complex geometry of the gray matter (Figs. 6–9). Note that in these images, the white matter (Figs. 6–9) has not been masked out. A number of features were revealed when the networks were considered at this spatial scale.

First, the two networks often contained regions that were closely juxtaposed along the cortical mantle, sometimes with sharp transitions between the networks (e.g., Figs. 6*C* and 7*C*). Second, the two networks showed few regions of overlap in all three 7T subjects, despite the low threshold of *z*(*r*) > 0.2, which permits weak correlations (<0.3) to be observed. Third, the two networks can occupy distinct regions deep within the same sulcus, sometimes on opposite banks a few millimeters apart (Fig. 9, *A*, *C*, *F*, *G*, and *H*). Fourth, regions of the two networks were sometimes interdigitated in complex ways, with sequential regions following the curvature of the gray matter (Fig. 9, *A*, *C*, *D*, and *F*). Fifth, a region belonging to one network can sit deep within the fundus and be surrounded on either side by regions belonging to the other network (e.g., Fig. 9*F*). There did not appear to be a clear relationship between the geometry of the gray matter and the location of the transition zone between the networks. It is not the case that a specific region of one network was always situated or always extended to a specific point along the sulcus, such as the fundus (see also Amunts et al. 1999). Rather, the network regions and their transitions were observed at different points in different sulci.

The above observations illustrate the extent to which the two networks are closely intertwined within the cortical anatomy and suggest, within the resolution and sensitivity available in these data, that the networks often occupy nonoverlapping regions of the cortical sheet. Furthermore, there did not appear to be a specific anatomical location where the two networks showed overlap consistently across subjects, as might be expected if a region was a reliable site of convergence. As an example, note how the overlap regions in the dorsomedial prefrontal cortex of S5 (Fig. 8) are not present in S3 and S4 in (Figs. 6 and 7).

### Consistent Spatial Relationships at High Resolution

Consistent spatial relationships were present when the networks were considered in all three high-resolution individuals together (Figs. 6–8). Such details may be informative for techniques that seek to record from or stimulate specific networks (e.g., intracranial methods). Below we describe some of these features. Sulcal and gyral anatomy were determined by comparison with the Duvernoy (1999) reference atlas. The observations are intended to provide approximate anatomical landmarks.

#### Posteromedial cortex.

In the posterior midline (Figs. 6*C*, 7*C*, and 8*C*), the two networks occupied sequential regions in an interdigitated fashion, beginning in the cingulate sulcus at the most anterior portion of the PCC and descending through the subparietal sulcus into ventral PCC and RSC. The callosal sulcus (thin light gray band circling the corpus callosum in Figs. 6–8, *top*) was spared by both networks, even in the region immediately posterior to the splenium (note that in S3 in Fig. 6, *network A* skirts the RSC border immediately inferior to where the callosal sulcus terminates). The interdigitated network regions extended into the marginal segment of the cingulate sulcus, containing a *network A* region at the junction between the subparietal and cingulate sulci in all three subjects. More ventrally, the interdigitated regions also extended into the transverse parietal sulcus (which originates near to the subparietal sulcus and ascends to the dorsal precuneus, bisecting the medial parietal lobe), containing a large *network B* region at or immediately ventral to the junction with the subparietal sulci in all three subjects.

A consistent feature was that *network A* occupied the most ventral regions of posteromedial cortex, whereas *network B* generally occupied dorsal regions of the PCC. Specifically, a large region of *network A* was found to extend along the parietal bank of the parieto-occipital fissure in all three participants. *Network A* also consistently occupied more dorsal regions in the posterior midline (i.e., the dorsal precuneus). In this way, *network A* could be seen to surround on three sides the *network B* region in the PCC.

Finally, following the cingulate sulcus rostrally, side-by-side regions could be seen extending as far as the middle cingulate cortex (Vogt 2009) in all three subjects, confirming the previous observation of this zone as a site of juxtaposition between the two networks (see Fig. 3, “zone 6” in Braga and Buckner 2017).

#### Medial temporal lobe.

An anatomical difference previously observed between the two distributed networks was that *network A* exhibited strong correlation with a region of the MTL at or near the posterior PHC, whereas no such evidence was found for *network B* (even in more a focused analysis; see Fig. S2 in Braga and Buckner 2017). In the present high-resolution data, we explored the functional connectivity of the MTL in the volume in more detail, to ascertain if further insights could be gained about the relation of this region to *networks A* and *B*.

**Fig. 10.**
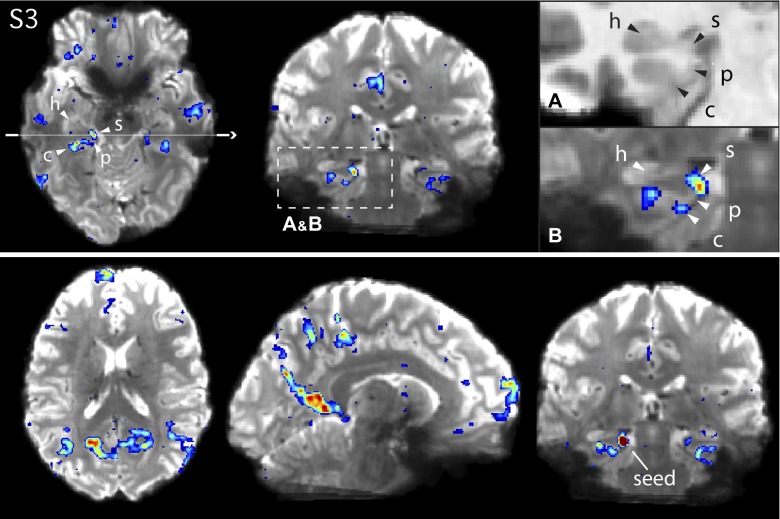
*Network A* shows connectivity with circumscribed regions of the medial temporal lobe (MTL) in *subject 3* (S3). *Top*: *network A* in S3, defined using a left dorsolateral prefrontal cortex seed (not shown). Arrows on the axial slice (*left*) and *insets* denote the approximate locations of the hippocampus proper (h), the subiculum (s), the parahippocampal cortex (p), and the contralateral sulcus (c). The thin white line denotes the position of the coronal slice. *Insets* show magnified images of the MTL in the coronal slice for the subject’s anatomical (T1 weighted) image (*A*), to display the white and gray matter anatomy clearly, and the functional connectivity map for *network A* overlaid onto the subject’s mean blood oxygenation level-dependent (BOLD) image (*B*). The mean BOLD underlay allows regions to be visualized in relation to the true BOLD anatomy given image distortion. Note that the arrows are placed at slightly different locations in *A* and *B* to match the anatomy in each image. *Bottom*: the functional connectivity map from a seed placed directly in the subiculum, which recapitulates the cortical components of *network A*, including the frontal pole near BA10 (see Fig. 6 for comparison).

*Network A* exhibited strong correlation with a region of posterior PHC in all three subjects. This region lay deep within the collateral sulcus (see *top left* axial views in Figs. 10 and 12) and extended along the posterior-anterior axis of this sulcus, beginning just anterior to the atrium of the lateral ventricles and extending rostrally. It was difficult to ascertain the most anterior extent of this PHC region due to this section of the MTL being affected by magnetic susceptibility artifacts from the nearby ear canals and sinus (Fig. 5; Ojemann et al. 1997). Thus it is unresolved whether the *network A* region extends further or, alternatively, whether there is a representation of *network B* in this zone that includes entorhinal cortex.

**Fig. 11.**
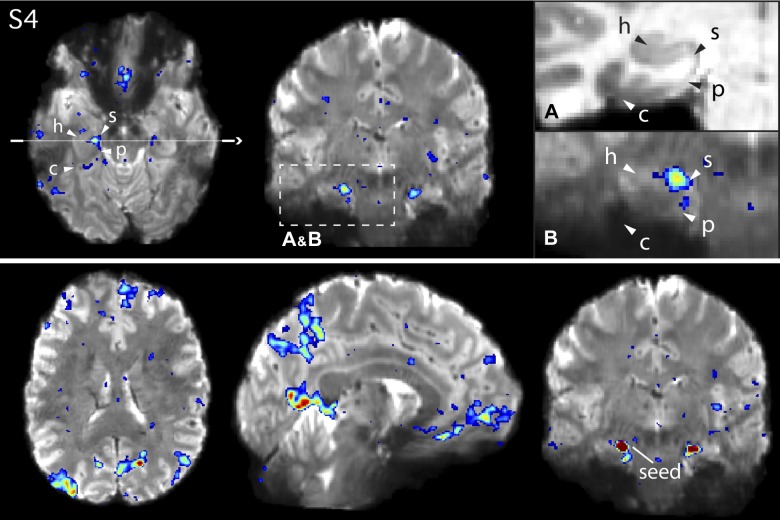
*Network A* shows functional connectivity with circumscribed regions of the medial temporal lobe (MTL) in *subject 4* (S4). *Top*: *network A* in S4, defined using a left dorsolateral prefrontal cortex seed (not shown). Arrows denote the approximate locations of the hippocampus proper (h), the subiculum (s), the parahippocampal cortex (p), and the contralateral sulcus (c). *Insets* show magnified images of the MTL in the coronal slice for the subject’s anatomical (T1 weighted) image (*A*), to display the white and gray matter anatomy clearly, and the functional connectivity map for *network A* overlaid onto the subject’s mean blood oxygenation level-dependent image (*B*). *Bottom*: the functional connectivity map from a seed placed directly in the subiculum, which recapitulates the cortical components of *network A*. See Fig. 7 for comparison with similar views of the network defined from the dorsolateral prefrontal cortex seed.

**Fig. 12.**
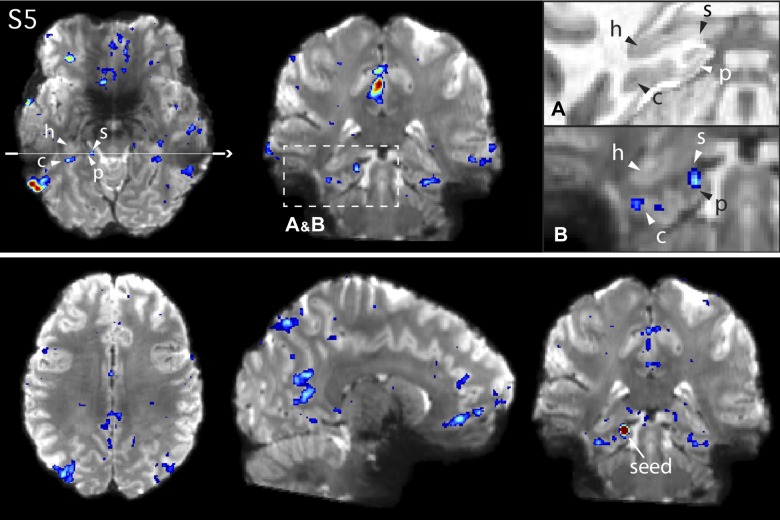
*Network A* shows functional connectivity with circumscribed regions of the medial temporal lobe (MTL) in *subject 5* (S5). *Top*: *network A* in S5, defined using a left dorsolateral prefrontal cortex seed (not shown). Arrows denote the approximate locations of the hippocampus proper (h), the subiculum (s), the parahippocampal cortex (p), and the contralateral sulcus (c). *Insets* show magnified images of the MTL in the coronal slice for the subject’s anatomical (T1 weighted) image (*A*), to display the white and gray matter anatomy clearly, and the functional connectivity map for *network A* overlaid onto the subject’s mean blood oxygenation level-dependent image (*B*). *Bottom*: the functional connectivity map from a seed placed directly in the subiculum, which recapitulates the cortical components of *network A*. See Fig. 8 for comparison with similar views of the network defined from the dorsolateral prefrontal cortex seed.

In all three participants, an additional region of *network A* could be resolved at or near the crown of the subiculum within the hippocampal formation itself, further demonstrating this network’s inclusion of traditional declarative memory structures. Panels *A* and *B* in Figs. 10–12 zoom in on the MTL in each subject on a coronal slice to highlight the anatomical details of this subicular region. The region can be observed near or at the crown of the subiculum, distinct from the PHC region in the collateral sulcus and further into the hippocampal formation. To confirm the validity of this region as a component of *network A*, a seed voxel was placed directly in the subiculum, and the resulting correlation map recapitulated the full and selective distribution of *network A* (Figs. 10–12, *bottom*). The subicular region was situated toward the middle of the anterior-posterior hippocampal axis, approximately where the MTL curves around the brain stem at the level where the raphe nuclei can be observed in the axial plane. Depending on the individual’s anatomy, the subicular and PHC region could be observed on the same axial and coronal slices (see Figs. 10 and 12).

The functional connectivity of the hippocampus was also explored at lower thresholds [*z*(*r*) ≈ 0.15]. Hints of small regions belonging to *networks A* and *B* could be observed at these thresholds, particularly in the subject that provided the most robust data (S3). However, in the remaining subjects, these hippocampal regions were suggestive only and could not be conclusively established. Further analyses of even higher resolution data and/or data with less artifact in the anterior MTL may be able to resolve regions within the hippocampal formation that show connectivity with the distributed association networks.

#### Medial prefrontal cortex.

The two distributed networks were closely interdigitated and occupied alternating regions in the MPFC (sagittal view in Figs. 6–8, *top*, and Fig. 6*D*). In general, *network B* occupied a large swath of MPFC in all subjects. *Network B* regions extended from the dorsal parts of the MPFC, descending rostrally along the midline and reaching to the most inferior portions of the MPFC. *Network A* regions were interdigitated with *network B* regions across the MPFC in a complex organization, though generally did not extend as dorsally or ventrally as *network B* regions. A *network A* region was identified in the most rostral portion of the MPFC, at or near the medial aspect of area 10 as originally demarcated by Brodmann (Fig. 86 in Brodmann 2006; see updated architectonic descriptions in Öngür et al. 2003; Petrides and Pandya 1994).

The ventral portion of the anterior midline is a site of projections from limbic structures, including the amygdala (Öngür and Price 2000) and hippocampal formation (Rosene and Van Hoesen 1977). An important observation is that a *network B* region was located at a more ventral region of the anterior midline than *network A*. This was surprising because, in contrast with *network A*, *network B* does not show a strong correlation with posterior PHC, although coupling to anterior MTL regions where artifact and signal loss are prominent cannot be ruled out. *Network A* occupied a dorsal subgenual region, whereas *network B* occupied more inferior regions extending rostrally along ventral MPFC (sagittal view in Fig. 6, *top*, and Figs. 6*D*, 7*D*, and 8*D*; see Fig. 5 in Córcoles-Parada et al. 2017 for sulcal variations in this region). Although this region is susceptible to signal drop out in fMRI, a subgenual region belonging to *network A* was resolved in all three individuals (sagittal view in Figs. 6–8, *top*).

## DISCUSSION

The canonical DN comprises at least two parallel distributed networks when defined within the individual (Braga and Buckner 2017). The two networks, referred to as *networks A* and *B*, display closely neighboring regions in multiple zones of the cortex and follow a general motif characteristic of the association networks (Buckner and Margulies in press; Doucet et al. 2011; Goldman-Rakic 1988; Margulies et al. 2016; Power et al. 2011; Yeo et al. 2011). In the present study we resolved the two networks using high-resolution scanning at 7T and describe a number of novel features with particular emphasis on anterior and posterior midline regions previously hypothesized to be zones of convergence.

### Parallel Distributed Networks Within the Default Network Are Replicated

Two networks within the canonical group-defined DN were reliably observed in five new individuals (Figs. 2, 3, and 6–8) and identified using both manually selected seed-based correlation and data-driven clustering, on the surface and in the volume (Fig. 4). These results demonstrate that the broad definition of the networks is not dependent on idiosyncratic analysis choices or due to experimenter bias. Furthermore, the two networks were identified in high-resolution data at 7T (Figs. 6–8). These collective results offer strong evidence that *networks A* and *B* represent stable features of brain organization.

### Parallel Distributed Networks Are Closely Juxtaposed Along the Cortical Ribbon

Anatomical details of the two networks were resolved by analyzing high spatial resolution data. At high spatial resolution, the two networks followed the geometry of the gray matter (Figs. 6–9) and were often closely juxtaposed along the cortical mantle, displaying sharp transitions between regions (see magnified images in Figs. 6–9, and especially Fig. 9, *E* and *G*). One individual (S5; Fig. 8) generally showed more overlap than the others.

Some degree of overlap is expected due to the limited resolution (e.g., partial volume effects) and spatial smoothing. However, it is notable that as we increased the effective resolution, going from 3T to 7T, the emergent picture was one of more separation rather than more overlap. In some locations, minimal overlap was observed between the two networks, despite the use of a liberal threshold and no restrictions being placed on a voxel being correlated with both seeds. The anatomical locations that did contain overlap were not obviously consistent across the three individuals examined (e.g., compare Figs. 6*C* and 8*C* with Fig. 7*C*). The general appearance was that overlap, when present, was near to the edges where two otherwise distinct regions were located.

In particular, the separation of the two networks along the posterior and anterior midline in the present data was striking (e.g., Figs. 6*C*, 7*C*, and 8*C*). Prior studies have emphasized that the posterior midline may be a hub or core that interacts with multiple distinct subnetworks or distributed systems (Andrews-Hanna et al. 2010; Braga et al. 2013; Buckner et al. 2009; Tomasi and Volkow 2010, 2014; see also Braga and Leech 2015; Power et al. 2013). The present findings raise the alternative possibility that the networks contain distinct regions situated near to one another along the complex cortical geometry of the midline. In group-averaged data, these regions are blurred together, giving the appearance of a network-general or task-general convergence zone.

One prior task-based study of individuals at 7T noted a complex organization of domain-specific subdivisions at or near the precuneus that responded differentially to tasks demanding orientation to space, time, and person (Peer et al. 2015). Analysis of their detailed results in light of the present observations yields an interesting point of concordance. In their study, they identified a region in the center of the precuneus responding most strongly to “person-oriented” emphasis that was surrounded by three adjacent regions responding to “space-oriented” emphasis (see Fig. 4*A* in Peer et al. 2015). This topography is similar to the presently observed organization that differentiates *network A* from *network B*. Specifically, in three of their individual subjects, a clear juxtaposition of the person-oriented response can be seen surrounded by distinct space-oriented responses (see *subjects 4*, *6*, and *12* in Supplementary Fig. 1 in Peer et al. 2015). A parsimonious explanation for the collective results may be that their task paradigm was differentially activating *network A* for the space-oriented condition and *network B* for the person-oriented condition.

In the present data, no consistent relationship was noticed between the gross cortical anatomy (i.e., the gyral and sulcal folds) and the position of transition zones between regions across individuals. In some cases, the juxtaposition between the two networks was simple, with two neighboring regions sitting side by side along the cortex (e.g., Fig. 9, *C* and *G*). In other cases, the two networks displayed a complex interdigitation, with alternating regions following the cortical ribbon in an irregular pattern (e.g., Figs. 6*C* and 9*F*).

Interdigitation of closely positioned regions has been previously observed in the cortex at various spatial scales. Anatomical projections from prefrontal and parietal association zones converge on adjacent columns of the cortical mantle in the anterior and posterior midline (see Figs. 4 and 6 in Selemon and Goldman-Rakic 1988). Alternating ocular dominance bands permeate striate cortex that display sharp boundaries between bands (Hubel and Wiesel 1979). Further along the visual hierarchy, face-responsive regions of the inferior temporal lobe appear as a set of noncontiguous islands (the “Fusiform Face Archipelago”; Kanwisher 2010; Moeller et al. 2008; Spiridon et al. 2006) surrounded by regions that respond preferentially to other visual stimulus categories (e.g., colors, scenes, or objects; Lafer-Sousa and Conway 2013; Lafer-Sousa et al. 2016). While it is not presently possible to link our fMRI findings with these anatomical features, it is nonetheless intriguing that the distributed parallel arrangement of closely juxtaposed and interdigitated functional regions echoes the distributed network features observed in direct anatomical studies. One possibility is that such features emerge because they are all outcomes of competitive activity-dependent processes that sculpt cortical organization during early development.

### Distinct Regions Are Revealed Within the Medial Temporal Lobe

*Network A* is robustly coupled to a specific region of the MTL at or near to the posterior PHC, whereas *network B* is not (Braga and Buckner 2017). The present analyses confirmed this distinction repeatedly (see surfaces in Fig. 4). In the high spatial resolution data, the PHC region participating in *network A* was resolved to a portion of the contralateral sulcus in all three 7T subjects (Figs. 10 and 12 for S3 and S5; other subject not shown).

The high-resolution data allowed us to resolve a key additional anatomical finding. A distinct region of the MTL, inside the hippocampal formation, at or near to the subiculum, also showed functional connectivity with *network A* (Figs. 10–12). In one participant (S4; Fig. 11), this region was positioned beyond the crest of the parahippocampal gyrus, extending to the beginning of the hippocampal sulcus. The subiculum is a source of direct cortical efferents to the RSC as well as subgenual prefrontal cortices (Rosene and Van Hoesen 1977). These subicular projections may form part of the larger distributed network we delineate here using functional connectivity (see Figs. 10–12, *bottom*, which show the network defined from a seed placed in the subiculum).

In the present data we were also able to detect hints of smaller regions within the hippocampus of one subject, but these regions displayed low correlations with *network A* and were not reliably observed across individuals. Further exploration of the MTL at higher resolution using sequences optimized to avoid signal loss in the anterior portions of the MTL will be required to resolve details and determine whether there are components of *network B* within the anterior MTL. It is an open question whether *networks A* and *B*, or other networks, have components in the anterior MTL regions poorly sampled in the present data (and previously analyzed in Braga and Buckner 2017).

### Technical Considerations of Fine-Scale Functional Architecture

The fractionation of the default network into parallel networks was possible because of focus on the individual rather than group-averaged data. Prior work has shown that, even after spatial normalization, the location of functional regions can differ between individuals on the order of centimeters (e.g., see Fig. 6 in Steinmetz and Seitz 1991; Fig. 12 in Rajkowska and Goldman-Rakic 1995; Clark et al. 1996; Henssen et al. 2016). In some cases, we were able to resolve distinct regions within the same sulcus, millimeters apart (Fig. 9*C*). With even a modest amount of additional blurring across or within individuals, it is likely that these distinctions would be lost. Thus our results illustrate the utility of focusing on individuals, not as a means to reveal individual differences but as an approach to maintain as much spatial information as possible and allow functionally specialized circumscribed regions to be sampled accordingly (see also Braga and Buckner 2017; Fedorenko et al. 2012; Gordon et al. 2017; Huth et al. 2016; Laumann et al. 2015; Michalka et al. 2015).

In addition to focusing on individuals, steps were taken to minimize spatial blur in the current analyses. BOLD data were acquired using 1.35- and 2.4-mm isotropic voxels, preprocessed and resampled to 1-mm resolution using a single interpolation step, and smoothed minimally at 2- to 3-mm FWHM. It is likely that these steps contributed to the ability to find the present distinctions. However, high spatial resolution data are not always available, particularly in studies aimed at collecting data from larger cohorts.

Recent parcellation strategies have shown that spatial priors can aid the definition of detailed network structures (e.g., Chong et al. 2017; Hacker et al. 2013; Kong et al. 2018; Wang et al. 2015; see also Bzdok et al. 2015; Leech et al. 2012). It is possible that high-resolution network maps from intensively sampled individuals, such as those we define here, might be used as priors to guide the parcellation of brain regions in new individuals. This may allow the network distinctions made here to be targeted in subjects that have data acquired at lower resolution and quantity.

**Fig. 13.**
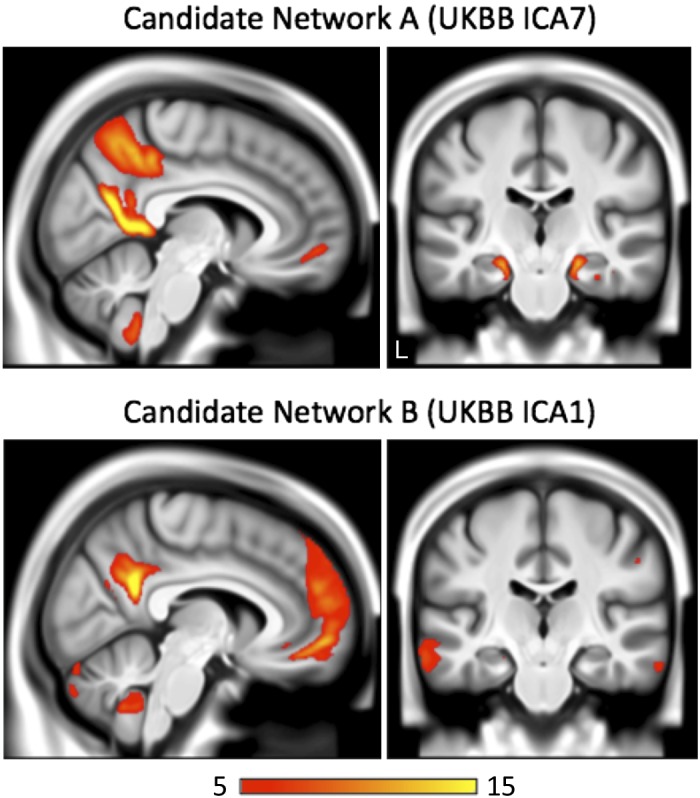
Network separation in population brain imaging data from 4,181 UK Biobank subjects. Shown are network estimates from two components (of 21 non-noise components) from an independent component analysis (ICA) of the UK Biobank neuroimaging data (Miller et al. 2016). *Top*: sagittal and coronal images from network *component 7* (UKBB ICA7). This network component has distributed features that parallel the within-individual estimates of *network A*, including regions within the posterior parahippocampus and distinct ventral and dorsal regions of the posterior midline. *Bottom*: sections from network *component 1* (UKBB ICA1). This network component has features aligned more with *network B*, including a posterior midline response between the separate *network A* components and much less prominent involvement of the posterior parahippocampal cortex. Although these ICA networks, derived from group-averaged data, do not align in all details with the individualized estimates, the detected parallel features suggest that distinctions between *networks A* and *B* can be probed to some degree in existing large-scale data sets.

Of interest to this possibility, guided by the hypothesis that *networks A* and *B* are distinct networks, one can find network structure in the UK Biobank data that may reflect this separation. The UK Biobank is a large-scale population-based study that is acquiring multimodal neuroimaging data on as many as 100,000 individuals (Sudlow et al. 2015). Given the pressure for efficiency, only a single resting-state functional run of 6 min 10 s is acquired in each individual for network analysis (Alfaro-Almagro et al. 2018; Miller et al. 2016). In the openly available network estimates from the first 4,181 individuals, there are two distinct networks that have many parallels with *networks A* and *B* as described here (Fig. 13 constructed from the 3D- maps browser at https://www.fmrib.ox.ac.uk/ukbiobank; Miller et al. 2016; see also Kernbach et al. 2018). Specifically, their “component #1” shares features with *network B* and their “component #7” with *network A*, including differential involvement of the posterior parahippocampal cortex and the differences in spatial organization around the posterior midline. Another example of such spatial distinctions in group-averaged data from the Human Connectome Project can be seen in Fig. 2 of Kong et al. (2018). Strategic use of priors may be able to refine such group-average estimates.

There is reason to suspect that smaller network regions remain unresolved even in the present data. In addition to the hints of small regions in the hippocampus discussed above, other findings were at the edge of detection in the subject that provided the best data. Although these regions included few voxels, their spatial arrangement suggested an actual biological origin. In particular, small alternating side-by-side and bilateral regions belonging to *networks A* and *B* were observed within the callosal sulcus extending from beneath the middle cingulate gyrus to the subgenual zone (not shown). These small regions also displayed low correlations and were not reliably observed in the other two high-resolution subjects. These observations are described here only to illustrate the possibility that additional analyses at higher resolution may resolve finer scale regions, particularly in deeper cortical and subcortical zones. Put simply, we are certain to still be missing important structure, even at the level of macroscale organization, that sits beyond our present resolution, data quality, and analysis strategies.

### Some Thoughts on the Anatomical Basis of the Observed Networks

The functional correlations utilized here to assess network organization depend on indirect hemodynamic measures of regional neuronal activity (Kwong et al. 1992; Ogawa et al. 1992) and are sensitive to physiological noise and motion, acquisition task state, and details of analytical processing (Buckner et al. 2013; Fox and Raichle 2007; Murphy et al. 2013; Power et al. 2014; Smith et al. 2013; Van Dijk et al. 2010). There are thus many steps and factors between anatomical organization and the present observations. Nonetheless, specific observations suggest that the observed networks may be related to underlying anatomical organization.

**Fig. 14.**
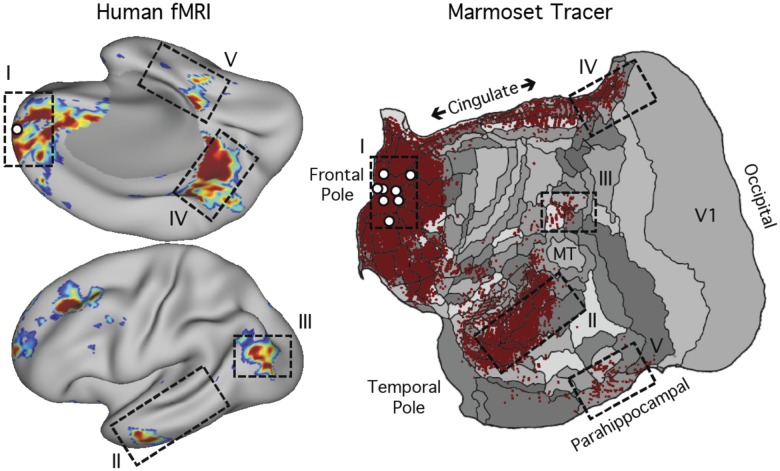
Human functional connectivity network estimates converge with marmoset tract tracing data. *Left*: the functional connectivity estimate of *network A* (DN-A) for a single seed region (white circle) placed into frontopolar cortex at or near Brodmann area 10 in *subject 1*. The medial view (*top*) is positioned to illustrate its continuity with the lateral view (*bottom*) to better appreciate similarities with the marmoset (see also macaque data presented in Goldman-Rakic 1988). Dashed rectangles labeled with Roman numerals I–V highlight 5 of the main zones of DN-A. *Right*: a flat map of marmoset cortex with retrograde label patterns from 8 separate frontopolar tracer injections (in A9, A10, and A11; see Buckner and Margulies in press; Burman et al. 2011). The red label displays the summation of the 8 cases. Lines on the flat map demarcate areal borders (Majka et al. 2016). Areas MT and V1 are labeled for reference. Dashed rectangles labeled with Roman numerals I–V highlight the estimated homologs of the 5 main zones of DN-A in the human. Note that the marmoset possesses a monosynaptically connected anatomical network that shows many parallels to the human DN-A identified with functional connectivity, including projections from parahippocampal cortex (*zone V*), posterior parietal cortex (*zone III*), and posterior cingulate (*zone IV*).

First, the broad network organization observed here in humans is similar to patterns observed from monosynaptic tract tracing studies in both macaque (Binder et al. 2009; Buckner et al. 2008; Margulies et al. 2009) and marmoset (Buckner and Margulies in press; Burman et al. 2011). To illustrate this key point, Fig. 14 displays the functional connectivity map for a seed region in frontopolar cortex within *network A* of S1 plotted next to a summary of marmoset tracer injections in frontopolar area A10 and adjacent areas A9 and A11. The marmoset data come from the openly available Marmoset Brain Architecture Project (http://www.marmosetbrain.org; Majka et al. 2016) and are plotted as described in Buckner and Margulies (in press; code openly available at https://github.com/margulies/marmoset).

What is striking is that the multiple distributed regions of the human network are predicted by the marmoset monosynaptic long-range projections (see Buckner and Margulies in press for discussion). Of particular interest, the PHC projects to frontopolar cortex A10 in New World monkeys (Burman et al. 2011; Rosa et al. 2019). When multiple anterior and posterior tracer injections are examined in relation to one another, a distributed network emerges that has clear parallels to *network A* in terms of its wide distribution as well as its specificity (Buckner and Margulies in press). We suspect the human correlational data revealing *network A* reflect the constraints of long-range anatomical connections either directly or through polysynaptic cortical-subcortical anatomical circuits (e.g., thalamocortical). Because the last common ancestor between New World monkeys and humans lived ~45 million years ago (Perelman et al. 2011; Hedges et al. 2015), these results suggest an ancient anatomical circuit motif supports the DN. It is unclear whether monkeys possess homologs of both *network A* and *network B* or rather a single, less differentiated network, shared by our last common ancestor, that then became expanded and specialized in the human lineage (Buckner and Margulies in press; Liu et al. in press).

The observation that human networks estimated through functional correlation roughly correspond to monkey long-range anatomical circuits places the observations on stronger footing than if observed in isolation (see also Margulies et al. 2009; Vincent et al. 2007). In analyses of human neuroimaging data, it is possible to partition variation to get multiple decompositions of networks and organization (e.g., Arslan et al. 2018). At high dimensionality, especially in weighting of local connectivity gradients, the networks often split apart into distributed local fragments. Without constraints of convergent anatomical data, it is unclear how to meaningfully interpret the human neuroimaging estimates of organization given the many possible estimates for the same data. The correspondence with monkey anatomical tracing data suggests that the present network descriptions reflect a method-invariant neurobiological level of organization. That is, even if there are further details and features of local organization that are not captured here, what is captured likely reflects a meaningful estimate of one level of network organization.

A second point is that the local regions observed here as components of the distributed networks unlikely align in a simple way to traditional notions of brain areas, especially as defined by cyto- and myeloarchitectonics. Classical notions of brain areas and their boundaries, anchored on sensory areas such as V1 and MT, posit that areal transitions are delineated by convergent architectonic, functional, topographic, and connectivity differences (Kaas 1987; see Van Essen and Glasser 2018 for discussion). Some recent analyses of human neuroimaging data build cortical parcellations using models that assume transitions between adjacent areas differ simultaneously on multiple dimensions (Glasser et al. 2016; see Eickhoff et al. 2018 for discussion). This assumption is likely true for some areas, especially early sensory areas when connectivity patterns (fingerprints) are considered at the level of the whole area.^1^ However, the assumption is unlikely to be true for all regions of cortex, especially for association zones where specialization and pruning of long-range connections may persist for months or years after birth.

As one relevant example of the disconnect between observable architectonic boundaries and anatomical connectivity, the long-range projections from architectonically defined frontopolar areas in the marmoset divide caudal architectonic areas TE3 and TPO (Buckner and Margulies in press; see also Rosa and Tweedale 2005; Schaeffer et al. 2019 for relevant discussion). A key transition, between cortex supporting canonical sensory-motor function and transmodal association zones, is not predicted by contemporary architectonic analysis but is captured by analysis of long-range connectivity patterns. Although it is possible that more sophisticated architectonic description will bring the two forms of data into alignment, it is also plausible that architectonic transitions of the traditionally emphasized form may not align fully with the organization of distributed association networks. This may be because the specific spatial patterns forming long-range networks arise through protracted competitive activity-dependent processes that shape and refine during hierarchical stages of development (for relevant discussion see Arcaro and Livingstone 2017; Buckner and Krienen 2013). It will be interesting to see advancement of analytical approaches that seek to unify different levels of organization across spatial scales (e.g., Schaeffer et al. 2019) and across modalities (e.g., Glasser et al. 2016). However, models should be open to the possibility that architectonic boundaries may not align with long-range connectivity in the adult brain in any simple manner for all zones of cortex.

### Conclusions

The present analyses extend our understanding of the fractionation of the DN into multiple parallel, distributed networks. The juxtaposition of the two networks adjacent to one another along the cortical ribbon and within individual sulci underscores the usefulness of examining functional anatomy at the level of the individual and at sufficient resolution. Precision functional mapping could aid applied endeavors that seek to record from or stimulate specific networks (e.g., using intracranial methods) as well as those seeking to limit complications arising from surgical resection.

## GRANTS

R. M. Braga was supported by Wellcome Trust Grant 103980/Z/14/Z and NIH Pathway to Independence Award K99MH117226. M. C. Eldaief was supported by Mentored Patient-Oriented Career Development Award K23MH099413. This work was also supported by Kent and Liz Dauten, NIH Grants P50MH106435 and P41EB015896, and Shared Instrumentation Grants S10OD020039 and S10RR019371.

## DISCLOSURES

K. R. A. Van Dijk is currently employed by Pfizer. Pfizer had no input or influence on the study design, data collection, data analyses, or interpretation of results. The remaining authors declare no competing financial interests.

## AUTHOR CONTRIBUTIONS

R.M.B., K.R.A.V.D., J.R.P., M.C.E., and R.L.B. conceived and designed research; K.R.A.V.D., J.R.P., M.C.E., and R.L.B. performed experiments; R.M.B., K.R.A.V.D., and R.L.B. analyzed data; R.M.B. and R.L.B. interpreted results of experiments; R.M.B. and R.L.B. prepared figures; R.M.B. and R.L.B. drafted manuscript; R.M.B., K.R.A.V.D., J.R.P., M.C.E., and R.L.B. edited and revised manuscript; R.M.B., K.R.A.V.D., J.R.P., M.C.E., and R.L.B. approved final version of manuscript.

## Footnotes

1 Here we refer to area-to-area connectivity defined as the systematic patterns of connections an area has to multiple other areas (Felleman and Van Essen 1991; Kaas 1987; Passingham et al. 2002). Local gradients of connectivity within areas, such as observed across central-to-peripheral projection zones in visual cortex, are also prominent features of connectional anatomy that can influence network estimates based on functional connectivity (Buckner and Yeo 2014).
